# Sedentary work and breast cancer risk: A systematic review and meta‐analysis

**DOI:** 10.1002/1348-9585.12239

**Published:** 2021-06-23

**Authors:** Jongin Lee, JaeYong Lee, Dong‐Wook Lee, Hyoung‐Ryoul Kim, Mo‐Yeol Kang

**Affiliations:** ^1^ Department of Occupational and Environmental Medicine Seoul St. Mary's Hospital College of Medicine The Catholic University of Korea Seoul Republic of Korea; ^2^ Department of Preventive Medicine College of Medicine Seoul National University Seoul Republic of Korea

**Keywords:** breast neoplasms, meta‐analysis, sedentary behavior, sedentary work, systematic review

## Abstract

**Objectives:**

This systematic review and meta‐analysis aimed to assess sedentary work's contribution to breast cancer risk quantitatively using thorough research articles.

**Methods:**

We performed a meta‐analysis using a registered protocol in PROSPERO (registration number: CRD42020204629). Literature from PubMed, Embase, and Cochrane involving sedentary work and breast cancer risk was reviewed. We calculated the overall pooled risk ratios (RRs) and 95% CI with a random‐effect model from the included studies. Furthermore, we performed stratified analyses by characteristics of studies.

**Results:**

Thirty‐one studies (13 cohort studies and 18 case‐control studies) were included in the analysis. The overall effect of the pooled analysis was an RR of 1.16 (95% CI 1.08‐1.23). The results were 1.20 (95% CI 1.10‐1.30) and 1.12 (95% CI 1.02‐1.23) for cohort and case‐control studies. The effect of sedentary work did not seem to be consistently attenuated by controlling body mass index, menopausal status, or experience of hormone replacement therapy.

**Conclusion:**

The results from this meta‐analysis suggest that sedentary behavior within the occupational domain was associated with a 15.5% increased risk of breast cancer. It is essential to reduce the sedentary time spent at work and to secure time for leisure‐time physical activity among sedentary workers as a primary preventive measure.

## 1 INTRODUCTION

Breast cancer is one of the most common cancers and the leading cause of cancer‐related deaths in women worldwide. There were 2.93 million incident cases and an estimated 0.63 million deaths from breast cancer globally in 2018, which significantly burdened the public health system.^1^ Although early detection and screening techniques have improved gradually, breast cancer incidence has been stable since 2004.

Previous epidemiological studies have shown that the incidence of breast cancer is associated with various risk factors, such as diet, obesity and weight gain, alcohol intake, tobacco smoke, prolonged hormone therapy after menopause, and use of oral contraceptives.^2^ The aging world's population, a marked increase in life expectancy, and a rapid tendency to adopt a Westernized lifestyle, including low fertility rates, sedentarism, and short breastfeeding periods, contribute to the accumulation of risk factors known to be associated with breast cancer. These factors contribute to the continual increase in the global burden of this cancer.^3^ Therefore, a public health priority is to identify environmental or lifestyle factors whose modifications could reduce breast cancer incidence.

An increase in sitting time accompanied by a decrease in physical activity levels in adults. Sedentary behavior is more widespread in modern life, and hence, people spend 50%‐60% of their waking time (7.7 h) sitting every day, and this number may continue to rise.^4^ Over the past decade, health consequences have been of increasing interest to the public. For example, it has been suggested that increased sitting time in daily life is associated with the risk of weight gain,^5^ obesity,^6^ Type 2 diabetes, coronary heart disease,^7^ and even cancer.^8^

Breast cancer is an obesity‐related type of cancer, and sedentary behavior and physical inactivity are known risk factors for it. The results of a meta‐analysis integrated from 21 observational studies with 34 reports showed that sedentary behavior was found to increase the risk of breast cancer (pooled odds ratio [OR] with a 95% confidence interval [CI] of 1.08 and 1.04‐1.13).^8^ However, this meta‐analysis only identified 12 studies on sedentary behavior in the occupation domain, and the evidence related to it has not been thoroughly assessed. Because the relatively larger proportion of time spent for occupation by working‐aged adults, it is important to ascertain if and by how much sedentary behavior in occupational domains influence the risks of breast cancers. Given the missing studies in the previous meta‐analysis and additional recent literature, an improved analysis needs a clear understanding of the effect of sedentary work on breast cancer risk. Therefore, this systematic review and meta‐analysis aimed to assess the contribution of sedentary work to breast cancer risk quantitatively.

## 2 MATERIALS AND METHODS

The protocol for this systematic review with meta‐analysis was registered in PROSPERO a priori. The review itself was conducted in accordance with the PRISMA statement guidelines.^9^

### 2.1 Searching and selection of studies

Three authors (JL, JYL, MYK) and a trained librarian searched the literature in PubMed, Embase, and Cochrane Library on January 11, 2020, using the following keywords: (“occupational physical activity,” “occupational physical inactivity,” “sedentary work,” “occupational sitting time,” “light work,” “occupational energy expenditure”) AND (“cancer,” “tumour,” “malignant,” “neoplasm,” “carcinoma”). Among the preliminary results, articles reporting the effects of breast cancer in English were used in this study. Two authors (JL and JYL) screened eligible studies per titles and abstracts. Furthermore, they selected available studies using the following inclusion criteria by reviewing all the articles' full texts. Three authors (JL, JYL and MYK) also examined the articles' reference lists from retrieved studies; studies not included in the preliminary search results were also included in the analysis.

### 2.2 Inclusion criteria

We included cohort and case‐control studies on breast cancer reporting effect sizes and 95% CI of “sedentary work” as an exposure variable. All studies with different study populations, for example, articles of postmenopausal women only or carcinoma in situ, were also included.

### 2.3 Extraction of data

From the included articles, we extracted the study name (first author and publication year), study design, country, the total number of participants, number of cases, sedentary work definition, comparison group definition, and effect sizes (odds ratios for case‐control studies and relative risks/hazard ratios for cohort studies) with 95% CI. Most of the studies used multiple levels of occupational physical activity. The basic principle to select effect sizes was to compare the least active group with the most active group. Some studies reported effect sizes of the occupationally active group compared with the sedentary group. In this case, we used reciprocal numbers of the effect sizes and confidence intervals of the comparison.

### 2.4 Quality assessment

The Newcastle‐Ottawa Scale is a tool widely used in the quality assessment of the meta‐analysis of observational studies.^10^ Three authors independently estimated the quality score using the Newcastle‐Ottawa Scale. Afterward, they resolved disagreements by discussion. Studies were classified into two categories: fine (six stars or more) and coarse (five stars or less).

### 2.5 Statistical analysis

We calculated the overall pooled risk ratios (RRs) and 95% CI with a random‐effect model from the included studies. Furthermore, we performed stratified analyses by publication year, study location, quality assessment, sedentary work definition, and adjusted variables, including body mass index (BMI), recreational or leisure‐time physical activity (LTPA), and experience of hormone replacement therapy (HRT). Some studies have reported divided results by menopausal status, estrogen/progesterone receptor, or stage (in situ vs invasive). We also performed subgroup meta‐analyses using these stratified results.

The heterogeneity among the studies was assessed by *I* ^2^ statistics following these criteria: *I*² of <25%, 25%‐50%, and <75% was set to low, moderate, and high, respectively. Begg's and Egger's tests were used to evaluate publication bias.^11^, ^12^ A visual inspection was conducted using a funnel plot. We used R software (Vienna, Austria) with its “meta” package.^13^ All statistical tests were two‐sided. A *P*‐value of 0.05 and a 95% CI were considered statistically significant.

## 3 RESULTS

### 3.1 Study selection

**FIGURE 1:**
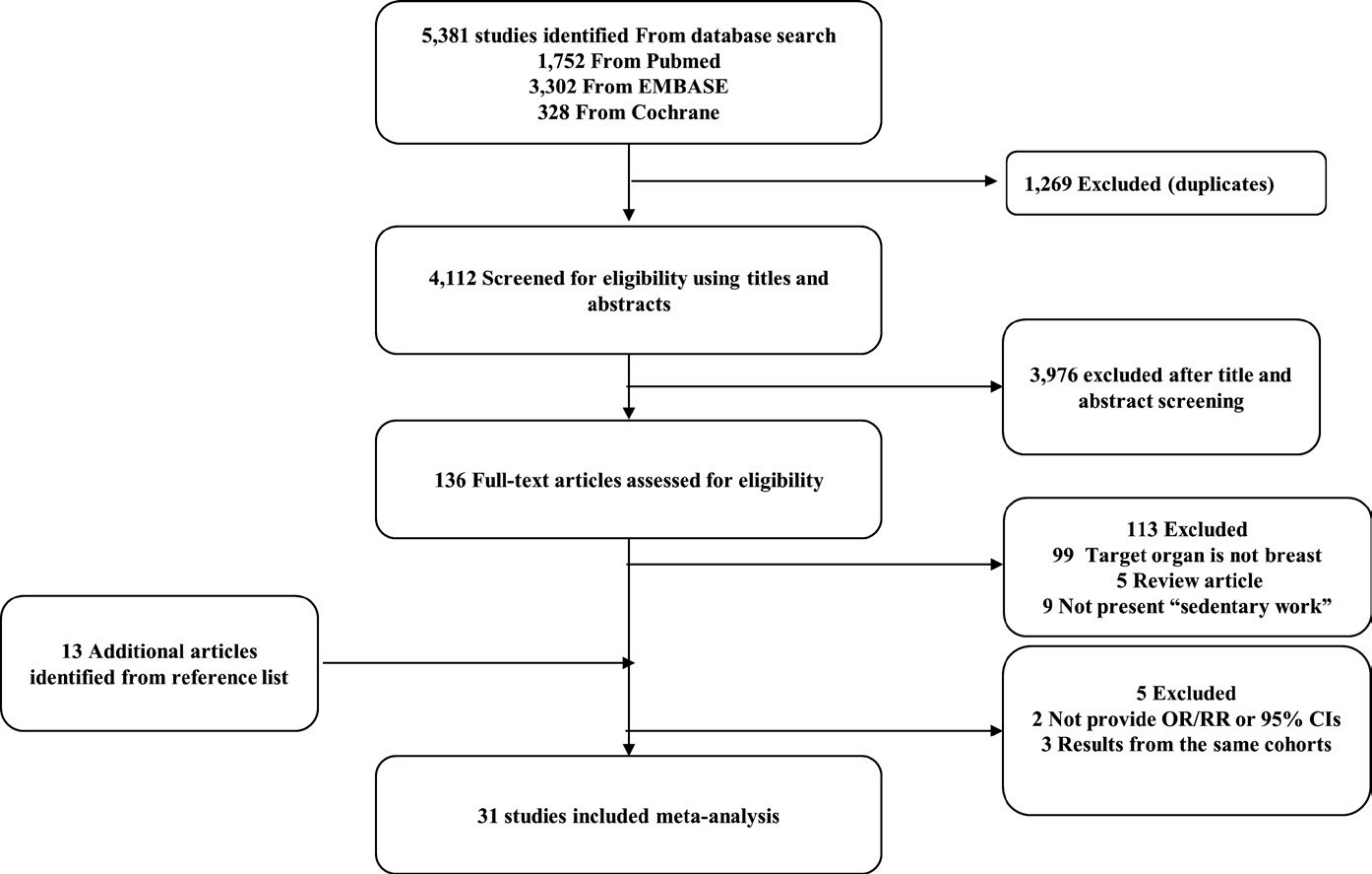
Flow diagram of processes for study selection in the meta‐analysis

Overall, we found 5381 studies regarding (occupational) physical activity and cancer by preliminary searching. We collected 136 studies eligible for the analysis between sedentary work and cancer after the removal of duplicates and screening of the abstract. Among them, 34 studies (16 cohort studies^14^, ^15^, ^16^, ^17^, ^18^, ^19^, ^20^, ^21^, ^22^, ^23^, ^24^, ^25^, ^26^, ^27^, ^28^, ^29^ and 18 case‐control studies^30^, ^31^, ^32^, ^33^, ^34^, ^35^, ^36^, ^37^, ^38^, ^39^, ^40^, ^41^, ^42^, ^43^, ^44^, ^45^, ^46^, ^47^) met our inclusion criteria. Because three and two studies were from the same cohort (European Prospective Investigation into Cancer [EPIC]^20^, ^27^, ^29^ and Black Women's Health Study,^24^, ^27^ respectively), we selected studies that could represent the overlapped population.^24^, ^29^ Finally, 31 studies (13 cohort studies and 18 case‐control studies) were included in the analysis (Figure 1).

### 3.2 Characteristics of the studies

Most of the selected studies did not use the same definition for sedentary work. Three main types of definitions were used: types of work, sitting time at work, and the metabolic equivalent of task (MET). We assessed five cohort studies and seven case‐control studies of fine quality. Studies with fine quality tended to have fewer participants. However, this tendency was not absolute (Table 1).

**TABLE 1:** Characteristics of studies included in the meta‐analysis

Study name	Design	Country	Number of participants	Number of cases	Comparison	Definition of sedentary work/definition of comparison group	Adjustment of confounders	Quality assessment
Thune et al^14^	Cohort	Norway	25 624	351	Type	Four types of work: sedentary/heavy manual labor	Age at entry, body mass index, height, county of residence, and number of children	Fine
Moradi et al^22^	Cohort	Sweden	1 940 510	51 520	Type	Five levels of physical activity: sedentary/high or very high	Age (by 5‐year intervals), calendar year of follow‐up by year, place of residence, socioeconomic status (in the analysis of occupational physical activity), or physical activity (in the analysis of socioeconomic status)	Coarse
Dirx et al^15^	Cohort	The Netherlands	62 537	1208	Sitting time	Sitting time per day (h): 6‐8 h/<2 h	Age, age at menarche, age at menopause, benign breast disease, parity, age at first birth, maternal breast carcinoma, breast carcinoma in sister(s), education, height, and baseline alcohol and energy intake	Coarse
Moradi et al^23^	Cohort	Sweden	9539	506	Type	Three types of activity: sedentary/strenuous	Age	Fine
Rintala et al^26^	Cohort	Finland	680 000	5721	Type	Five classes of tasks: sitting/heavy task	Social class and reproductive factors	Coarse
George et al^17^	Cohort	United States	97 039	3436	Sitting time	Occupational activity: sitting all day/heavy lifting or carrying	Age, energy intake, recreational physical activity, parity, menopausal hormone, number of breast biopsies, smoking, alcohol intake, race, education	Coarse
Pronk et al^25^	Cohort	China	73 049	717	Sitting time	Average sitting time: ≥4 h/day/≤1.2 h/day	Age, education, family history of breast cancer, age at first birth, and number of pregnancies	Fine
Steindorf et al^28^	Cohort	Mixed (Europe)	257 805	8034	Type	Three types of activity: sedentary/manual and heavy manual	BMI, age at first period, age at first full‐term pregnancy, number of full‐term pregnancies, breastfeeding, ever oral contraceptive, menopausal status, age at menopause, use of hormone replacement therapy, alcohol consumption, smoking status, level of school attained; other types of physical activity	Coarse
Ekenga et al^16^	Cohort	United States	47 649	1798	Type	Occupational history: mostly sitting/active	Race/ethnicity, education, income, parity, age at menopause, BMI, recreational physical activity, and work at night	Coarse
Masala et al^21^	Cohort	Italy	15 010	672	Type	Three types of activity: sedentary/manual	Education, number of children, age at menarche, non‐alcohol energy intake, current use of HRT, smoking status, and total vegetables consumption, physical activity, alcohol consumption, and anthropometric measures	Coarse
Nomura et al^24^	Cohort	United States	46 734	2041	Sitting time	Sitting time/day (h): 5+ h/day/<1 h/day	Age, geographic region of residence, BMI, education, recreational physical activity, caloric intake, parity, age at menarche, menopausal hormone use, oral contraceptive use, family history of breast cancer, mammogram, smoking	Coarse
Johnsson et al^19^	Cohort	Sweden	29 524	1506	Type	Three types of baseline occupation data: sedentary/non‐sedentary	Age at inclusion, occupation, competitive sports, family history of breast cancer, age at birth of first child, age at menarche, HRT, BMI	Fine
Ihira et al^18^	Cohort	Japan	33 307	3807	Sitting time	Occupational sitting time: ≥7 h/day/1‐3 h/day	Age, area, history of diabetes, smoking status, alcohol intake status, body mass index, coffee, walking time at work, strenuous time at work, moderate‐to‐vigorous physical activity time in leisure time, type of job, and total working hours	Fine
Coogan et al^33^	Case‐control	United States	11 646	4863	Type	Four types of work: sedentary/heavy	Age, state, BMI, benign breast disease, family history of breast cancer, menopausal status, age at menarche, parity, age at first birth, education, physical activity during ages 14‐22, and alcohol consumption	Fine
Levi et al^38^	Case‐control	Switzerland	620	246	Type	Three grades of intensity of work: low/high	Age, education, age at menarche, age at first birth, number of births, menopausal status, age at menopause, calorie intake, previous benign breast disease, and history of breast cancer in first‐degree relatives	Coarse
Coogan and Aschengrau^32^	Case‐control	United States	903	233	Type	Exclusively sedentary work/exclusively medium/heavy	Vital status, education, and total duration of working years.	Fine
Verloop et al^45^	Case‐control	The Netherlands	1836	918	Type	>80% sedentary work/≤80% sedentary work	Family history of breast cancer, education, smoking, occupational activity, and the other recreational physical activity variables	Coarse
Moradi et al^41^	Case‐control	Sweden	6802	3347	Type	Five levels of physical activity: sedentary/high or very high	Age, age at menarche, parity and age at first birth, BMI, height, use of hormone replacement therapy, age at menopause, and use of oral contraceptives	Fine
Matthews et al^40^	Case‐control	China	3015	1459	Sitting time	Longest sitting time/short sitting time	Age, education, household income, first‐degree family history of breast cancer, history of breast fibroadenoma, age at menarche, age at first live birth, and age at menopause	Fine
Friedenreich et al^35^	Case‐control	Canada	2470	1233	MET	MET‐h/week/year/≥61.8	Current age, waist‐hip ratio, educational level, ever use of hormone replacement therapy, ever diagnosed with benign breast disease, first‐degree family history of breast cancer, ever alcohol consumption, current cigarette smoker, and other types of activity	Coarse
Dorn et al^34^	Case‐control	United States	1550	740	MET	Percent of work years/moderate + jobs: 0%/100%	Age, education, age of menarche, relative with breast cancer, benign breast disease, BMI and age first pregnancy	Coarse
Steindorf et al^44^	Case‐control	Germany	1246	360	Type	MET‐h/week in occupation: 0.0/35.1‐170.5	First‐degree family history of breast cancer, number of full‐term pregnancies, height, change in body mass index between age 20 and 30 years, total months of breastfeeding, and mean daily alcohol consumption	Coarse
Kruk et al^37^	Case‐control	Poland	822	257	Type	Three types of activity: sedentary/medium	BMI, age at menarche, sport and recreational activities, intake of vegetables and fruits and experience of stress	Coarse
Yang et al^46^	Case‐control	United States	1095	501	Type	Job category: sedentary only/active blue collar	Education, migration history, parity, family history of breast cancer, menopausal status, average MET h/week for all recreational activities, and soy intake during adolescence and adult life	Coarse
Sprague et al^43^	Case‐control	United States	15 710	8080	MET	MET‐h/week: 0/>100	Age, state, mammography, menopausal status, family history of breast cancer, parity, age at first birth, age at menarche, age at menopause, postmenopausal hormone use, education, alcohol, BMI, and weight change since age 18	Fine
Peplonska et al^42^	Case‐control	Poland	4502	2176	MET	MET‐h/week of lifetime: <11.3/>47.8	Age, study site, education, BMI, age at menarche, menopausal status, age at menopause (in postmenopausal women), number of full‐term births, age at first full‐term birth, breastfeeding, family history of breast cancer, and previous screening mammography	Coarse
Kruk^36^	Case‐control	Poland	1943	858	Type	Four activity level: sedentary/high	Age, BMI, lifetime recreational physical activity, age at menarche, age at first full‐term pregnancy, parity, months of breastfeeding, intake of vitamins, active smoking, passive smoking, family history of breast cancer	Coarse
Cohen et al^31^	Case‐control	United States	2730	546	Sitting time	Sitting at work, h/day: >3/none	Age, race, menopausal status, education, household income, BMI at age 21, cigarette smoking, ever use of hormone replacement therapy, parity, age at menarche, first‐degree family history of breast cancer, and having health insurance	Coarse
Lynch et al^39^	Case‐control	Canada	2452	1222	Sitting time	Occupational sitting time (h/week/year): ≥7.3/0	Current age, educational level, lifetime total physical activity, caloric intake, ever alcohol consumption, smoking status, waist‐hip ratio, menopausal status, total number of mammograms, first‐degree family history of breast cancer, ever use of hormone replacement therapy, number of children breastfed	Fine
Yen et al^47^	Case‐control	Malaysia	243	121	MET	MET‐h/week/year: <102.0/>140.2	Current age, menopausal status, secondary smoke exposure and 6‐month history of breastfeeding	Coarse
Boyle et al^30^	Case‐control	Australia Canada	4294	1762	Type	Sit‐JEM: sedentary/sit‐JEM75	Age, study location, education, ethnicity, recreational physical activity in early adulthood, body mass index in early adulthood, number of births, breastfeeding status, shift work status. and years worked in an active occupation	Fine

### 3.3 Overall analysis

**FIGURE 2:**
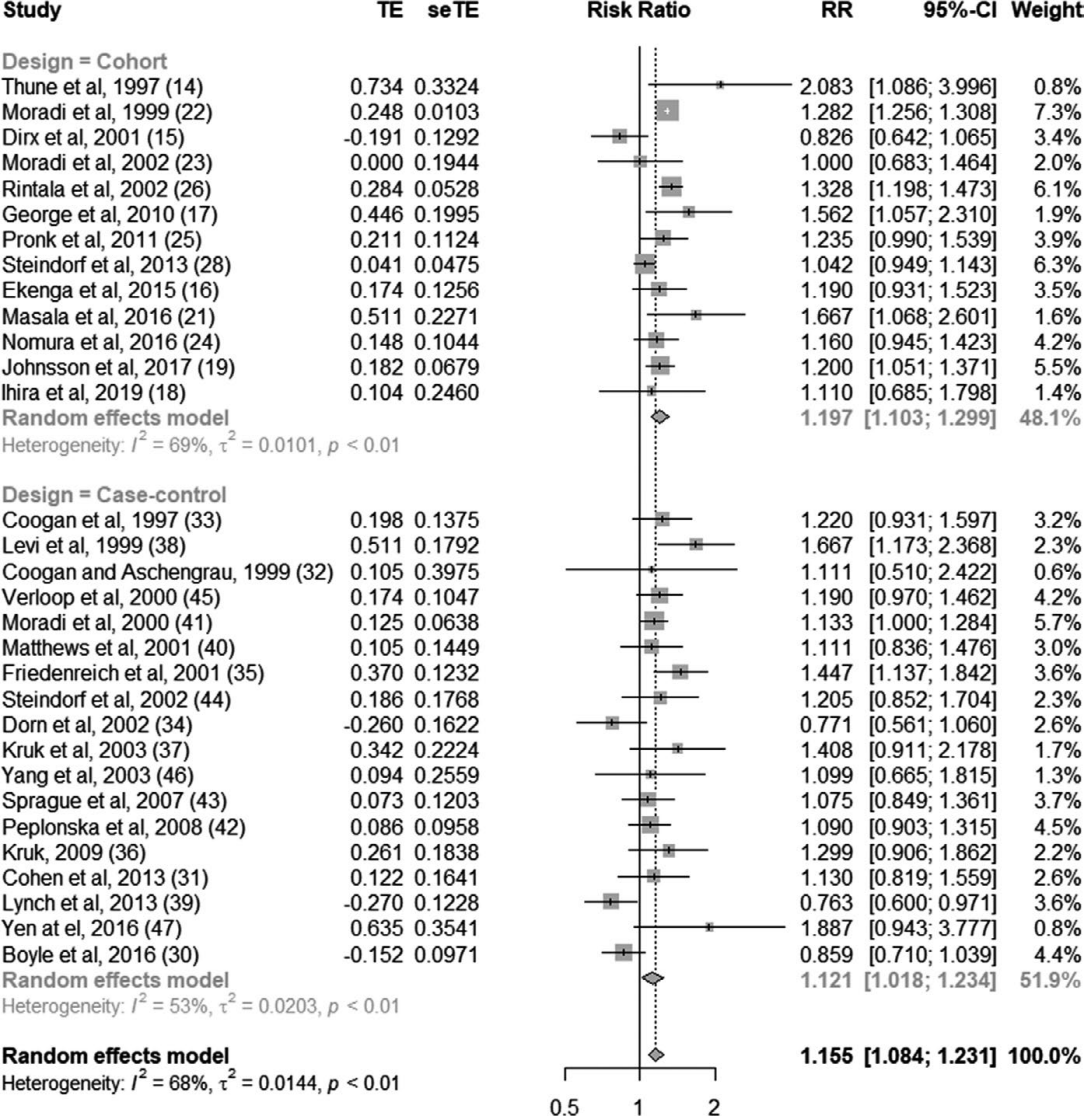
Forest plot of risk ratios between sedentary work and breast cancer, divided by study design

The overall pooled estimates were an RR of 1.16 (95% CI 1.08‐1.23). The effects were 1.20 (95% CI 1.10‐1.30) and 1.12 (95% CI 1.02‐1.23) for cohort and case‐control studies, respectively (Figure 2). The pooled estimates were almost the same between the two design groups, as no difference was observed between them (*P* for difference = 0.31). The overall *I* ^2^ score was 68%, which showed high heterogeneity (*P* < .01). The *I* ^2^ scores by study design were 69% and 53% for cohort studies and case‐control studies, respectively, with high heterogeneity (*P* < .01).

**FIGURE 3:**
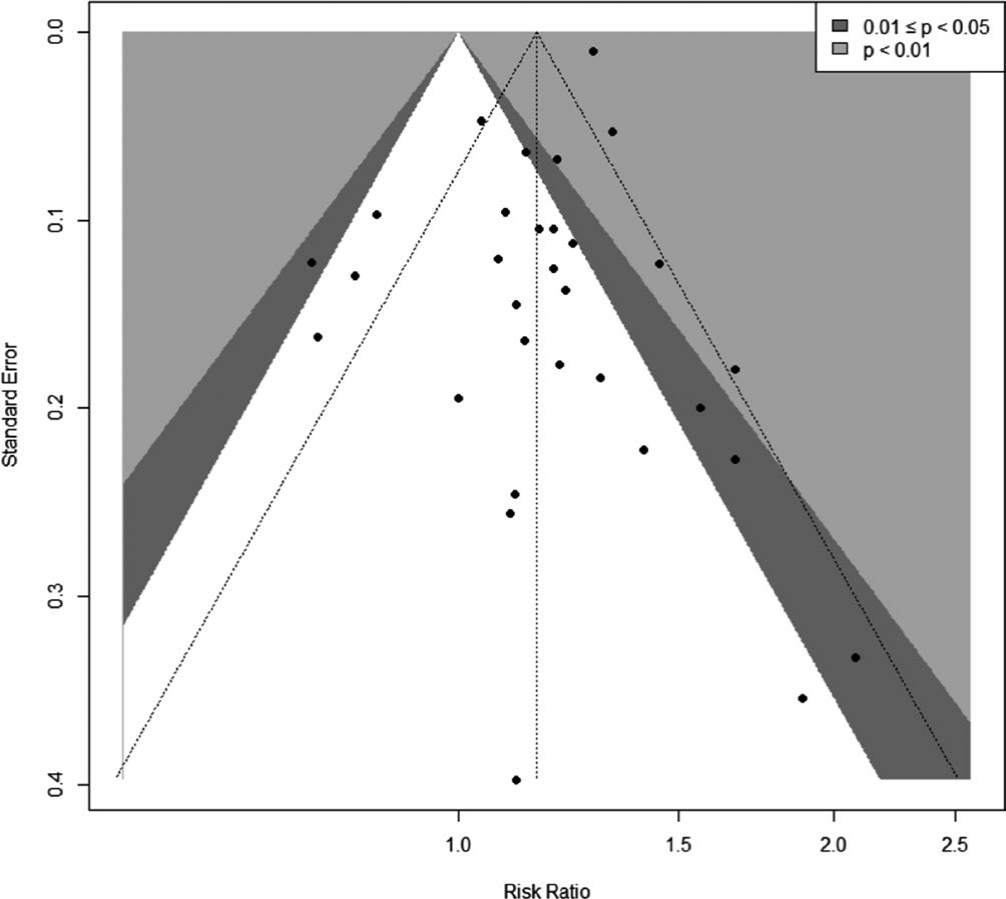
Funnel plot for the studies between sedentary work and breast cancer

**FIGURE 4:**
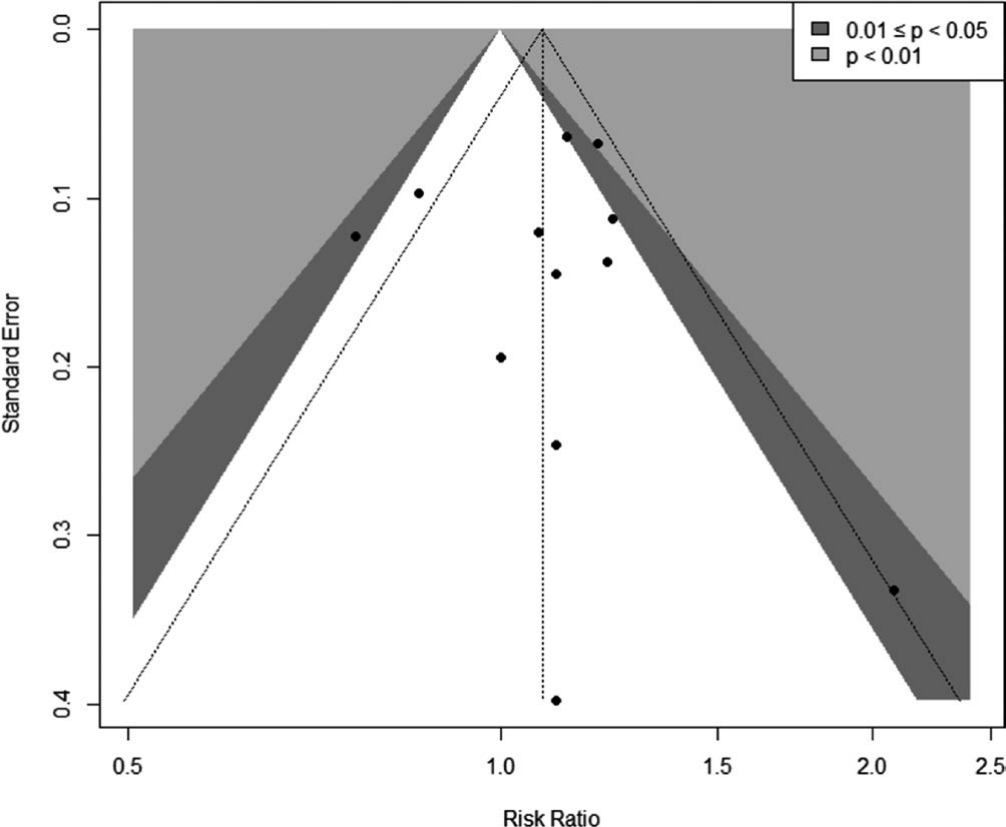
Funnel plot for the studies between sedentary work and breast cancer, fine‐quality studies only

The visual inspection of the funnel plot showed asymmetry (Figure 3). The *P*‐values for Begg's and Egger's tests were 0.03 and 0.04, respectively. This publication bias was diluted in the sensitivity analysis limited to the studies of high quality, although visual asymmetry was still seen in the sensitivity analysis limited to fine‐quality studies (Figure 4). However, the *P*‐values for Begg's and Egger's tests were 0.78 and 0.96, respectively. Because of the differences in study design, we also generated funnel plots by study design (cohort and case‐control, Figures S1 and S2). The *P*‐values for Begg's and Egger's tests were 0.46 and 031, respectively, for cohort studies and 0.16 and 0.34, respectively, for case‐control studies.

### 3.4 Subgroup analysis

All the studies assessed sedentary work using the following three methods (Table 2): classifying types of work, assessing daily sitting time at work, and calculating METs. Studies that assessed the type of work and METs showed significantly increased RRs (1.18 95% CI [1.09‐1.27] and 1.21 95% CI [1.04‐1.40], respectively). However, studies with sitting time did not (RR, 1.07; 95% CI, 0.91‐1.25). Studies with METs, which were all case‐control designs, showed low heterogeneity (*I* ^2^ = 30.7%, *P* = .22).

**TABLE 2:** Pooled risk of breast cancer according to sedentary work derived from subgroup analysis

Subgroup	Number of studies included	Pooled RR	*I* ^2^	*P*‐value for heterogeneity
Assessment for comparison				
Type of work	18	1.178 [1.090; 1.273]	70.0%	<.0001
Sitting time	8	1.069 [0.914; 1.250]	60.1%	.0143
MET	5	1.208 [1.042; 1.402]	30.7%	.2168
*P* value for subgroup difference = .4789				
Region				
Europe	15	1.168 [1.030; 1.324]	55.1%	<.0001
America	12	1.079 [0.945; 1.231]	61.7%	.0025
Asia	4	1.207 [1.029; 1.416]	0.0%	.5572
*P* value for subgroup difference = .2427				
Publication year				
Before 2010	19	1.187 [1.105; 1.275]	53.8%	.0026
After 2010	12	1.120 [1.004; 1.249]	61.6%	.3389
*P* value for subgroup difference = .3814				
Quality assessment^a^				
Fine	12	1.081 [0.974; 1.200]	51.6%	.0192
Course	19	1.202 [1.113; 1.297]	65.1%	<.0001
*P* value for subgroup difference = .1092				
Adjustment of confounder^b^				
BMI	18	1.114 [1.025; 1.210]	54.0%	.0034
LTPA	14	1.163 [1.068; 1.267]	68.9%	<.0001
HRT	10	1.144 [1.033; 1.267]	61.8%	.0051
Menopausal status				
Premenopausal	10	1.099 [0.968; 1.247]	10.7%	.3444
Postmenopausal	11	1.105 [0.968; 1.263]	67.8%	.0006
Not mentioned	19	1.212 [1.138‐1.290]	47.6%	.0113
*P* value for subgroup difference = .9487				
Cancer characteristics				
Carcinoma in situ only	3	1.045 [0.959; 1.139]	0.0%	.9527
Estrogen receptor positive	2	1.102 [0.971; 1.252]	0.0%	.8435
Estrogen receptor negative	2	1.162 [0.843; 1.602]	42.5%	.1874

Abbreviations: BMI, body mass index; HRT, hormone replacement therapy; LTPA, leisure‐time physical activity; MET, metabolic equivalent of task.

^a^ The cutoff value for quality assessment with Newcastle‐Ottawa Scale was six stars: fine (six stars or more) and coarse (five stars or less).

^b^ Subgroups of studies that had used confounders listed in the table were analyzed independently.

All studies from Europe and Asia showed significantly increased RRs (1.17 95% CI [1.03‐1.32] and 1.21 95% CI [1.03‐1.42], respectively). However, studies from America did not (RR, 1.08; 95% CI, 0.95‐1.23). All pooled RRs by publication year showed significant RRs (1.19 95% CI [1.11‐1.28] and 1.12 95% CI [1.00‐1.25], respectively). Pooled RR from coarse‐quality studies showed significant RR (1.20 95% CI [1.11‐1.30]), while the RR did not meet statistical significance among fine‐quality studies (1.08 95% CI [0.97‐1.20]).

All studies that adjusted BMI, LTPA, or HRT showed significant RRs. However, results from the subgroup analyses based on menopausal status (n = 10 for premenopausal and n = 11 for postmenopausal) or cancer characteristics (n = 3 for carcinoma in situ only, n = 2 for estrogen receptor‐positive only, and n = 2 for estrogen receptor‐negative only) did not yield significant results at all. The pooled result from studies that have not mentioned about menopausal status showed significantly increased risk (RR, 1.21 95% CI [1.14‐1.29]).

## 4 DISCUSSION

In this study, we quantitatively reviewed the existing observational epidemiologic evidence on the relationship between sedentary work and breast cancer risk. Compared with the previous meta‐analysis,^8^ we included 19 additional studies and explored the influence of the occupational domain of sedentary behavior in more depth. The findings from this systematic review and meta‐analysis suggest that sedentary behavior within the occupational domain was associated with a 15.5% increased risk of breast cancer, while previous meta‐analysis reported only showed 10% increased risk of breast cancer associated with occupational sedentary behavior.^8^ This may be because recently published studies have produced relatively higher risk estimates.^16^, ^18^, ^19^, ^21^, ^24^, ^47^

Several plausible biological mechanisms have been proposed to explain how sedentary behavior increases the risk of breast cancer, including the possible effect of sedentarism on adiposity, insulin resistance, systemic inflammation, sex hormones, and breast density. These are thought to contribute to the development and progression of breast cancer.^8^ Most of the available evidence implies the role of reducing energy expenditure with weight gain over time, leading to cancer development. Adiposity can promote carcinogenesis through several pathways, including elevated estrogen in postmenopausal women, insulin resistance, perturbation of the insulin‐like growth factor axis, and low‐grade systemic inflammation.^48^ In our meta‐analysis, however, the effect of sedentary work did not seem to be consistently attenuated by controlling BMI. Accumulating epidemiological evidence suggests that higher physical activity levels may lower the risk of certain types of cancers independent of BMI.^49^ For example, Reeves et al reported that overweight, the most apparent consequence of sedentary behavior, was an independently‐related breast cancer risk in postmenopausal women, suggesting that fat accumulated through sedentary behavior is an independent contributor to breast cancer and a mediator in other pathways.^50^ Nevertheless, the impact of sedentary behavior on cancer incidence, especially obesity‐related cancer, does not seem to be entirely adiposity‐independent to date. The potential role of the adiposity‐independent pathway on this association requires further clarification, as this knowledge can help provide a better interpretation of current knowledge in this specific area of interest.

Sex hormones, including estrogens, are associated with an increased risk of pre‐ and postmenopausal breast cancer.^51^ Sedentary behavior and physical inactivity have been hypothesized to influence the endogenous production of sex steroid hormones by altering menstrual cycle patterns and increasing body fat.^52^ However, adjustments for menopausal status or HRT did not significantly attenuate the association between sedentary work and breast cancer in our subgroup analyses, suggesting that sedentary work does not wholly exert its biological effects hormonal mechanisms.

We noted that the positive association between sedentary work and breast cancer was less pronounced among fine‐quality studies than others. The stronger association in low‐quality studies could arise from biases, such as selection bias, recall bias, misclassification, and confusion, which may have obliterated the true relationship in those studies. However, pooled estimates were almost the same between cohort and case‐control studies (1.20 and 1.12, respectively), although prospective cohort studies are less prone to healthy worker selection bias and recall bias than case‐control studies. In addition, we could not find statistical differences by assessment for comparison, publication year, and menopausal status. Likewise, the difference by study region was statistically insignificant, but studies from Asian countries showed slightly stronger associations of breast cancer risk with sedentary work, compared with that from American regions. We assumed that most of the population within these areas are of similar race and ethnicity. There may be inequities regarding social ort, cultural norms, or economic obligations across the study region. Based on the above findings, we propose that race and ethnicity should be considered important effect modifiers in the analysis while investigating the associations between risk of breast cancer and sedentary work in future studies.

This systematic review and meta‐analysis on the relationship between sedentary work and breast cancer risk is extensive and comprehensive. All existing scientific evidence from 31 epidemiological studies was included. Therefore, the results of meta‐analyses provide sufficiently reliable estimates of breast cancer risk associated with sedentary work. However, some methodological limitations of this study must be considered. First, there were variations across studies in the methods used to ascertain sedentary work as exposure, and categorization of sedentary work was highly heterogeneous; therefore, it was difficult to make direct comparisons between the included studies. Moreover, there are concerns regarding the validity and reliability of job title‐based and self‐reported engagement in sedentary work, which was likely to cause a recall bias and exposure misclassification. A recent Japanese research demonstrated that without real‐time feedback of individuals' current activity levels, subjective sedentary time might be underestimated compared with objective measurement of sedentary time.^53^ Hence, it is expected that these possibilities would bias the results toward the null. Even though there was moderate heterogeneity throughout the study, our subgroup analysis of study characteristics identified some causes of this heterogeneity, such as publication year and study region. Second, due to the limited number of studies reporting information for potential confounding factors (eg gene, race/ethnicity, following a healthy diet, having regular medical check‐ups, and hormone receptor status), we were unable to perform subgroup analyses based on most of these factors. Third, because we used the extreme categories of highest and lowest sedentariness levels as exposure measures, we were not able to investigate a dose‐response relationship. Finally, it is suspected that the associations observed in the meta‐analysis of published studies may suffer from publication bias because studies with null results tend not to be published. However, contour‐enhanced funnel plots showed that many insignificant results were included in our meta‐analysis, and there was no evidence for a separate test by study design and sensitivity analysis limited to fine‐quality studies. Furthermore, we only selected literature written in English, which may have resulted in a language or cultural bias.

In summary, this systematic review and meta‐analysis of observational epidemiologic studies with the most up‐to‐date evidence showed that sedentary work is significantly associated with breast cancer risk. This finding indicates that it is essential to reduce the sedentary time spent at work and to secure time for LTPA among sedentary workers as a primary preventive measure.

## DISCLAIMER

None.

## FUNDING STATEMENT

This research received no specific grant from any funding agency in the public, commercial, or not‐for‐profit sectors.

## DISCLOSURE

*Approval of the research protocol*: Ethics approval for the present study was not required because this was a review of articles that is free of personally identifiable information. *Informed Consent*: N/A. *Registry and the Registration No. of the study/trial*: N/A. *Animal Studies*: N/A. *Conflict of Interest*: N/A.

## AUTHOR CONTRIBUTIONS

J. Lee: Methodology, software, formal analysis, data curation and writing—original draft preparation. JY Lee: Methodology, validation, data curation. DW Lee: Interpretation and validation. HR Kim: Conceptualization, validation, and interpretation. MY Kang: Conceptualization, methodology, validation, interpretation, writing—original draft preparation.

## Supporting information

Figure S1. Funnel plot for the studies between sedentary work and breast cancer, cohort studies only.
Figure S2. Funnel plot for the studies between sedentary work and breast cancer, case‐control studies only.

## References

[1] Ferlay J , Ervik M , Lam F , et al. Global Cancer Observatory: Cancer Today. International Agency for Research on Cancer. 2018. https://gco.iarc.fr/today. Accessed December 16, 2020.

[2] American Cancer Society . Breast Cancer Facts & Figures 2019–2020. Atlanta, GA: American Cancer Society, Inc.; 2019.

[3] Matthews CE , George SM , Moore SC , et al. Amount of time spent in sedentary behaviors and cause‐specific mortality in US adults. Am J Clin Nutr. 2012;95(2):437‐445. 22218159 10.3945/ajcn.111.019620 PMC3260070

[4] Blanck HM , McCullough ML , Patel AV , et al. Sedentary behavior, recreational physical activity, and 7‐year weight gain among postmenopausal U.S. women. Obesity (Silver Spring). 2007;15(6):1578‐1588. 17557996 10.1038/oby.2007.187

[5] Wijndaele K , Healy GN , Dunstan DW , et al. Increased cardio‐metabolic risk is associated with increased TV viewing time. Med Sci Sports Exerc. 2010;42(8):1511‐1518. 20139784 10.1249/MSS.0b013e3181d322ac

[6] Grøntved A , Hu FB . Television viewing and risk of type 2 diabetes, cardiovascular disease, and all‐cause mortality: a meta‐analysis. JAMA. 2011;305(23):2448‐2455. 21673296 10.1001/jama.2011.812 PMC4324728

[7] Schmid D , Leitzmann MF . Television viewing and time spent sedentary in relation to cancer risk: a meta‐analysis. J Natl Cancer Inst. 2014;106(7):dju098. 24935969 10.1093/jnci/dju098

[8] Zhou Y , Zhao H , Peng C . Association of sedentary behavior with the risk of breast cancer in women: update meta‐analysis of observational studies. Ann Epidemiol. 2015;25(9):687‐697. 26099193 10.1016/j.annepidem.2015.05.007

[9] Moher D , Liberati A , Tetzlaff J , Altman DG . Preferred reporting items for systematic reviews and meta‐analyses: the PRISMA statement. PLoS Medicine. 2009;6(7):e1000097. 19621072 10.1371/journal.pmed.1000097 PMC2707599

[10] Wells GA , Shea B , O’Connell D , et al. The Newcastle‐Ottawa Scale (NOS) for Assessing the Quality of Nonrandomised Studies in Meta‐Analyses. Oxford; 2000. http://www.ohri.ca/programs/clinical_epidemiology/oxford.htm. Accessed October 1, 2008.

[11] Begg CB , Mazumdar M . Operating characteristics of a rank correlation test for publication bias. Biometrics. 1994;50(4):1088–1101. 7786990

[12] Egger M , Smith GD . Meta‐analysis bias in location and selection of studies. BMJ. 1998;316(7124):61‐66. 9451274 10.1136/bmj.316.7124.61 PMC2665334

[13] Schwarzer G . Meta: an R package for meta‐analysis. R News. 2007;7(3):40‐45.

[14] Thune I , Brenn T , Lund E , Gaard M . Physical activity and the risk of breast cancer. N Engl J Med. 1997;336(18):1269‐1275. 9113929 10.1056/NEJM199705013361801

[15] Dirx MJM , Voorrips LE , Goldbohm RA , van den Brandt PA . Baseline recreational physical activity, history of sports participation, and postmenopausal breast carcinoma risk in the Netherlands Cohort Study. Cancer. 2001;92(6):1638‐1649. 11745243 10.1002/1097-0142(20010915)92:6<1638::aid-cncr1490>3.0.co;2-q

[16] Ekenga CC , Parks CG , Sandler DP . A prospective study of occupational physical activity and breast cancer risk. Cancer Causes Control. 2015;26(12):1779‐1789. 26450605 10.1007/s10552-015-0671-8 PMC4664182

[17] George SM , Irwin ML , Matthews CE , et al. Beyond recreational physical activity: examining occupational and household activity, transportation activity, and sedentary behavior in relation to postmenopausal breast cancer risk. Am J Public Health. 2010;100(11):2288‐2295. 20864719 10.2105/AJPH.2009.180828 PMC2951936

[18] Ihira H , Sawada N , Yamaji T , et al. Occupational sitting time and subsequent risk of cancer: the Japan Public Health Center‐based Prospective Study. Cancer Sci. 2020;111(3):974‐984. 31925977 10.1111/cas.14304 PMC7060463

[19] Johnsson A , Broberg P , Johnsson A , Tornberg ÅB , Olsson H . Occupational sedentariness and breast cancer risk. Acta Oncol. 2017;56(1):75‐80. 27919198 10.1080/0284186X.2016.1262547

[20] Lahmann PH , Friedenreich C , Schuit AJ , et al. Physical activity and breast cancer risk: the European Prospective Investigation into Cancer and Nutrition. Cancer Epidemiol Biomarkers Prev. 2007;16(1):36‐42. 17179488 10.1158/1055-9965.EPI-06-0582

[21] Masala G , Bendinelli B , Assedi M , et al. Up to one‐third of breast cancer cases in post‐menopausal Mediterranean women might be avoided by modifying lifestyle habits: the EPIC Italy study. Breast Cancer Res Treat. 2017;161(2):311‐320. 27832394 10.1007/s10549-016-4047-x

[22] Moradi T , Adami HO , Bergström R , et al. Occupational physical activity and risk for breast cancer in a nationwide cohort study in Sweden. Cancer Causes Control. 1999;10(5):423‐430. 10530613 10.1023/a:1008922205665

[23] Moradi T , Adami H‐O , Ekbom A , et al. Physical activity and risk for breast cancer a prospective cohort study among Swedish twins. Int J Cancer. 2002;100(1):76‐81. 12115590 10.1002/ijc.10447

[24] Nomura SJO , Dash C , Rosenberg L , Palmer J , Adams‐Campbell LL . Sedentary time and breast cancer incidence in African American women. Cancer Causes Control. 2016;27(10):1239‐1252. 27632430 10.1007/s10552-016-0803-9 PMC5527706

[25] Pronk A , Ji B‐T , Shu X‐O , et al. Physical activity and breast cancer risk in Chinese women. Br J Cancer. 2011;105(9):1443‐1450. 21934685 10.1038/bjc.2011.370 PMC3241547

[26] Rintala PE , Pukkala E , Paakkulainen HT , Vihko VJ . Self‐experienced physical workload and risk of breast cancer. Scand J Work Environ Health. 2002;28(3):158‐162. 12109554 10.5271/sjweh.659

[27] Rosenberg L , Palmer JR , Bethea TN , et al. A prospective study of physical activity and breast cancer incidence in African‐American women. Cancer Epidemiol Biomarkers Prev. 2014;23(11):2522‐2531. 25103823 10.1158/1055-9965.EPI-14-0448 PMC4221421

[28] Steindorf K , Ritte R , Eomois P‐P , et al. Physical activity and risk of breast cancer overall and by hormone receptor status: the European prospective investigation into cancer and nutrition. Int J Cancer. 2013;132(7):1667‐1678. 22903273 10.1002/ijc.27778

[29] Steindorf K , Ritte R , Tjonneland A , et al. Prospective study on physical activity and risk of in situ breast cancer. Cancer Epidemiol Biomarkers Prev. 2012;21(12):2209‐2219. 23074288 10.1158/1055-9965.EPI-12-0961

[30] Boyle T , Fritschi L , Kobayashi LC , et al. Sedentary work and the risk of breast cancer in premenopausal and postmenopausal women: a pooled analysis of two case–control studies. Occup Environ Med. 2016;73(11):735‐741. 27540104 10.1136/oemed-2015-103537

[31] Cohen SS , Matthews CE , Bradshaw PT , et al. Sedentary behavior, physical activity, and likelihood of breast cancer among Black and White women: a report from the Southern Community Cohort Study. Cancer Prev Res (Phila). 2013;6(6):566‐576. 23576427 10.1158/1940-6207.CAPR-13-0045 PMC3703619

[32] Coogan PF , Aschengrau A . Occupational physical activity and breast cancer risk in the upper Cape Cod cancer incidence study. Am J Ind Med. 1999;36(2):279‐285. 10398936 10.1002/(sici)1097-0274(199908)36:2<279::aid-ajim7>3.0.co;2-7

[33] Coogan PF , Newcomb PA , Clapp RW , Trentham‐Dietz A, Baron JA , Longnecker MP . Physical activity in usual occupation and risk of breast cancer (United States). Cancer Causes Control. 1997;8(4):626‐631. 9242479 10.1023/a:1018402615206

[34] Dorn J , Vena J , Brasure J , Freudenheim J , Graham S . Lifetime physical activity and breast cancer risk in pre‐and postmenopausal women. Med Sci Sports Exerc. 2003;35(2):278‐285. 12569217 10.1249/01.MSS.0000048835.59454.8D

[35] Friedenreich CM , Bryant HE , Courneya KS . Case‐control study of lifetime physical activity and breast cancer risk. Am J Epidemiol. 2001;154(4):336‐347. 11495857 10.1093/aje/154.4.336

[36] Kruk J . Lifetime occupational physical activity and the risk of breast cancer: a case‐control study. Asian Pac J Cancer Prev. 2009;10(3):443‐448. 19640188

[37] Kruk J , Aboul‐Enein HY . Occupational physical activity and the risk of breast cancer. Cancer Detect Prev. 2003;27(3):187‐192. 12787725 10.1016/s0361-090x(03)00032-1

[38] Levi F , Pasche C , Lucchini F , La Vecchia C . Occupational and leisure time physical activity and the risk of breast cancer. Eur J Cancer. 1999;35(5):775‐778. 10505038 10.1016/s0959-8049(99)00051-9

[39] Lynch BM , Courneya KS , Friedenreich CM . A case‐control study of lifetime occupational sitting and likelihood of breast cancer. Cancer Causes Control. 2013;24(6):1257‐1262. 23526070 10.1007/s10552-013-0194-0

[40] Matthews CE , Shu X‐O , Jin F , et al. Lifetime physical activity and breast cancer risk in the Shanghai Breast Cancer Study. Br J Cancer. 2001;84(7):994‐1001. 11286483 10.1054/bjoc.2000.1671 PMC2363839

[41] Moradi T , Nyrén O , Zack M , Magnusson C , Persson I , Adami HO . Breast cancer risk and lifetime leisure‐time and occupational physical activity (Sweden). Cancer Causes Control. 2000;11(6):523‐531. 10880034 10.1023/a:1008900512471

[42] Peplonska B , Lissowska J , Hartman TJ , et al. Adulthood lifetime physical activity and breast cancer. Epidemiology. 2008;19(2):226‐236. 18277160 10.1097/EDE.0b013e3181633bfb

[43] Sprague BL , Trentham‐Dietz A , Newcomb PA , Titus‐Ernstoff L , Hampton JM , Egan KM . Lifetime recreational and occupational physical activity and risk of in situ and invasive breast cancer. Cancer Epidemiol Biomarkers Prev. 2007;16(2):236‐243. 17301255 10.1158/1055-9965.EPI-06-0713

[44] Steindorf K , Schmidt M , Kropp S , Chang‐Claude J . Case‐control study of physical activity and breast cancer risk among premenopausal women in Germany. Am J Epidemiol. 2003;157(2):121‐130. 12522019 10.1093/aje/kwf181

[45] Verloop J , Rookus MA , Van der Kooy K , van Leeuwen FE . Physical activity and breast cancer risk in women aged 20–54 years. J Natl Cancer Inst. 2000;92(2):128‐135. 10639514 10.1093/jnci/92.2.128

[46] Yang D , Bernstein L , Wu AH . Physical activity and breast cancer risk among Asian‐American women in Los Angeles: a case–control study. Cancer. 2003;97(10):2565‐2575. 12733156 10.1002/cncr.11364

[47] Yen SH , Knight A , Krishna M , Muda W , Rufai A . Lifetime physical activity and Breast Cancer a case‐control study in Kelantan. Malaysia Asian Pac J Cancer Prev. 2016;17(8):4083‐4088. 27644665

[48] Anderson AS , Key TJ , Norat T , et al. European code against cancer 4th edition: obesity, body fatness and cancer. Cancer Epidemiol. 2015;39(l 1):S34‐S45. 26205840 10.1016/j.canep.2015.01.017

[49] Hidayat K , Zhou H‐J , Shi B‐M . Influence of physical activity at a young age and lifetime physical activity on the risks of 3 obesity‐related cancers: systematic review and meta‐analysis of observational studies. Nutr Rev. 2020;78(1):1‐18. 10.1093/nutrit/nuz024 31393566

[50] Reeves GK , Pirie K , Beral V , Green J , Spencer E , Bull D . Cancer incidence and mortality in relation to body mass index in the Million Women Study: cohort study. BMJ. 2007;335(7630):1134. 17986716 10.1136/bmj.39367.495995.AE PMC2099519

[51] Endogenous Hormones and Breast Cancer Collaborative Group . Sex hormones and risk of breast cancer in premenopausal women: a collaborative reanalysis of individual participant data from seven prospective studies. Lancet Oncol. 2013;14(10):1009‐1019. 23890780 10.1016/S1470-2045(13)70301-2 PMC4056766

[52] Clague J , Bernstein L . Physical activity and cancer. Curr Oncol Rep. 2012;14(6):550‐558. 22945451 10.1007/s11912-012-0265-5 PMC3490043

[53] Yamamoto K , Ebara T , Matsuda F , et al. Can self‐monitoring mobile health apps reduce sedentary behavior? a randomized controlled trial. J Occup Health. 2020;62(1):e12159. 32845553 10.1002/1348-9585.12159 PMC7448798

