# Supplementary material for: Sedentary work and breast cancer risk: A systematic review and meta‐analysis
Source: J Occup Health. 2021 Jun 23;63(1):e12239. doi: 10.1002/1348-9585.12239 (PMC8221371; doi:10.1002/1348-9585.12239)
Supplement: Supplementary file 1 — Figure S1. Funnel plot for the studies between sedentary work and breast cancer, cohort studies only. Figure S2. Funnel plot for the studies between sedentary work and breast cancer, case‐control studies only. [file JOH2-63-e12239-s001.pdf]

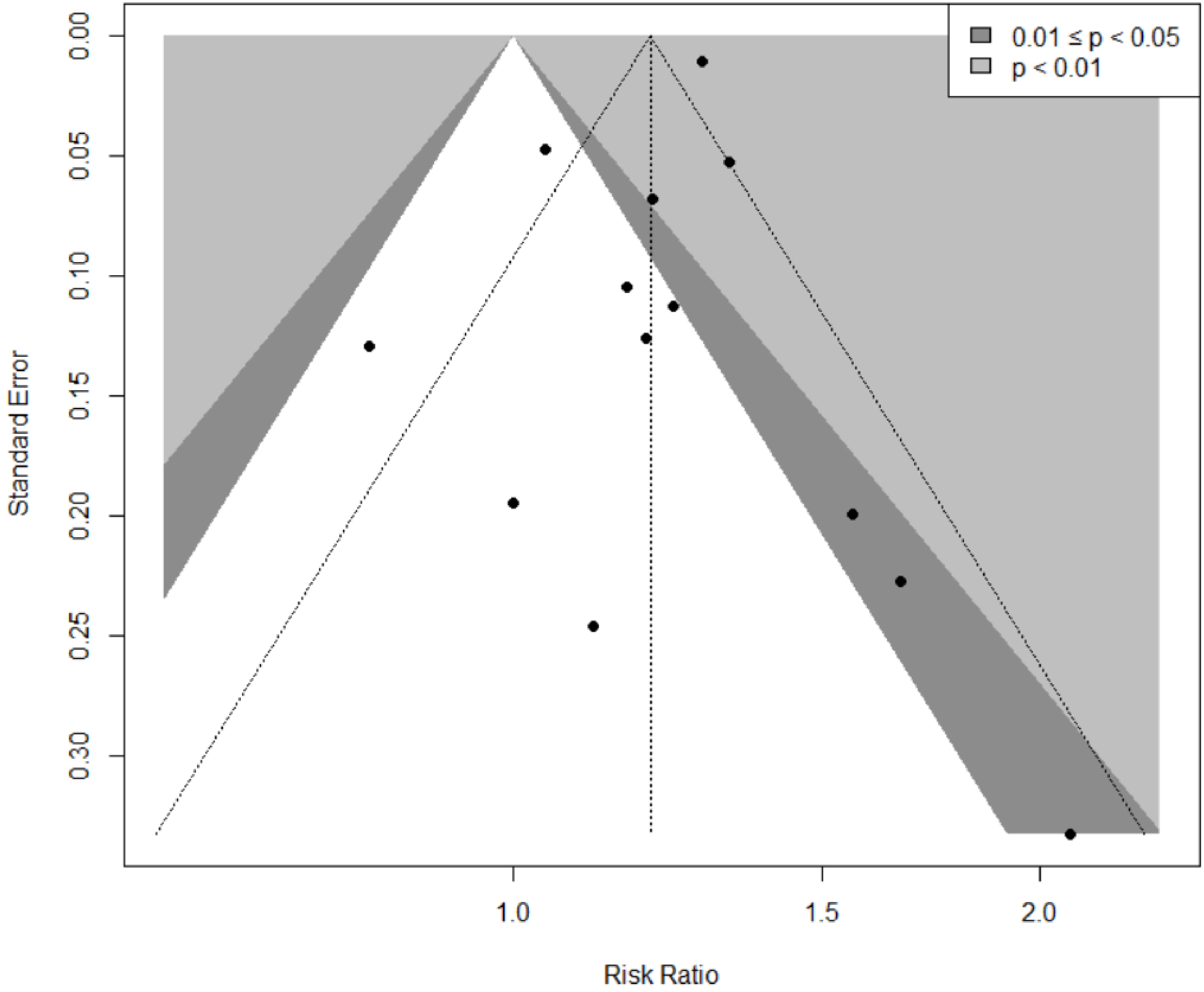

Supplementary figure 1. Funnel plot for the studies between sedentary work and breast cancer, cohort studies only

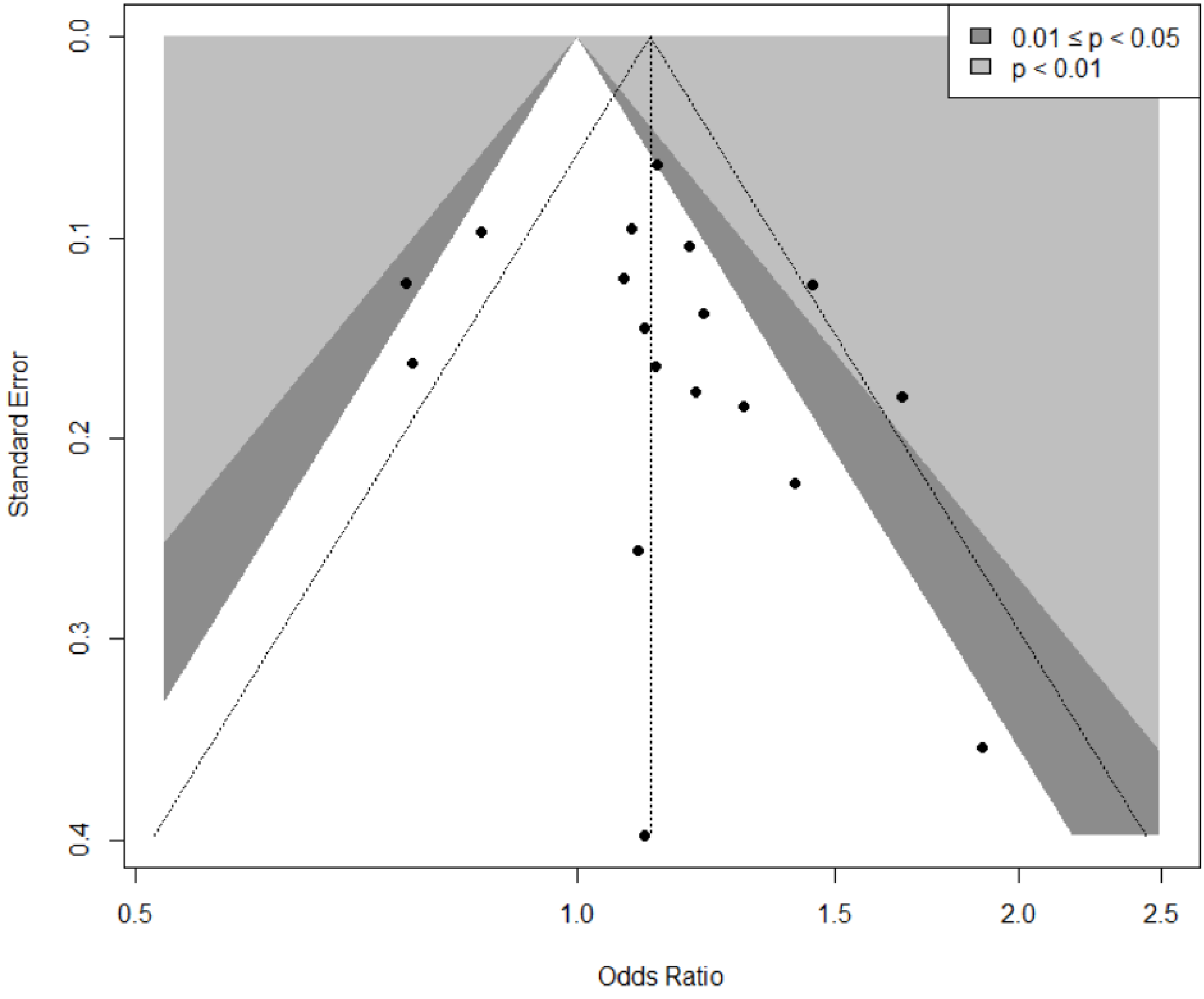

Supplementary figure 2. Funnel plot for the studies between sedentary work and breast cancer, case-control studies only
